# Modeling the Potential Global Distribution of Honeybee Pest, *Galleria mellonella* under Changing Climate

**DOI:** 10.3390/insects13050484

**Published:** 2022-05-22

**Authors:** Eslam M. Hosni, Areej A. Al-Khalaf, Mohamed G. Nasser, Hossam F. Abou-Shaara, Marwa H. Radwan

**Affiliations:** 1 Entomology Department, Faculty of Science, Ain Shams University, Cairo 11566, Egypt; marwahamdy@sci.asu.edu.eg; 2 Biology Department, College of Science, Princess Nourah Bint Abdulrahman University, Riyadh 11671, Saudi Arabia; aaalkhalaf@pnu.edu.sa; 3 Department of Plant Protection, Faculty of Agriculture, Damanhour University, Damanhour 22516, Egypt; hossam.farag@agr.dmu.edu.eg

**Keywords:** climate change, honeybee pests, Maxent, species distribution modeling, GWM

## Abstract

**Simple Summary:**

The greater wax moth (GWM) is a common pest of bee colonies throughout the world. This study highlighted the global habitat suitability of GWM using the statistical power of Maxent to model its current and future distribution under climate-change scenarios in 2050 and 2070. Our study shed light on the major environmental factors that manage the habitat suitability of GWM. To the best of our knowledge, this is the first modeling study of wax moths. In brief, this pest can cause severe indirect damage to the global honey market totalling millions of dollars; therefore, developing prompt monitoring or control strategies is advised.

**Abstract:**

Beekeeping is essential for the global food supply, yet honeybee health and hive numbers are increasingly threatened by habitat alteration, climate change, agrochemical overuse, pathogens, diseases, and insect pests. However, pests and diseases that have unknown spatial distribution and influences are blamed for diminishing honeybee colonies over the world. The greater wax moth (GWM), *Galleria mellonella*, is a pervasive pest of the honeybee, *Apis mellifera*. It has an international distribution that causes severe loss to the beekeeping industry. The GWM larvae burrow into the edge of unsealed cells that have pollen, bee brood, and honey through to the midrib of the wax comb. Burrowing larvae leave behind masses of webs that cause honey to leak out and entangle emerging bees, resulting in death by starvation, a phenomenon called galleriasis. In this study, the maximum entropy algorithm implemented in (Maxent) model was used to predict the global spatial distribution of GWM throughout the world. Two representative concentration pathways (RCPs) 2.6 and 8.5 of three global climate models (GCMs), were used to forecast the global distribution of GWM in 2050 and 2070. The Maxent models for GWM provided a high value of the Area Under Curve equal to 0.8 ± 0.001, which was a satisfactory result. Furthermore, True Skilled Statistics assured the perfection of the resultant models with a value equal to 0.7. These values indicated a significant correlation between the models and the ecology of the pest species. The models also showed a very high habitat suitability for the GWM in hot-spot honey exporting and importing countries. Furthermore, we extrapolated the economic impact of such pests in both feral and wild honeybee populations and consequently the global market of the honeybee industry.

## 1. Introduction

Over recent decades, apiculture has declined throughout the world as a result of decreasing numbers of managed honeybee (*Apis mellifera* L.) colonies [1,2]. To stimulate local apiculture and pollination, it is critical to make beekeeping a more appealing hobby and a less labor-intensive job [3]. Beekeeping is a significant human activity as well as an important component of the bioeconomy [4]. Furthermore, beekeeping provides several ecological services, many of which are important to humans [5,6,7,8,9]. Beekeeping products are used in food and medicine, and beekeepers profit financially [10]. For instance, wax is one of the most significant and beneficial beekeeping products, with applications in the pharmaceutical and cosmetics industries [11].

Increased pollinator density and diversity have a direct impact on agricultural productivity and can assist smallholder farmers to raise their output by a global average of 24%. It has a major impact on biodiversity and has favorable social effects [12]. Many negative factors currently affect global beekeeping, posing a threat to its long-term viability. Advancements in agriculture, degradation of natural ecosystems, pesticide contamination of bee forage lands, emergence of new bee diseases, honeybee pests, and climate change have all had a detrimental effect on beekeeping activities in recent decades [13].

Honeybee pests are known to cause considerable losses and spread viral infections that are difficult to eradicate and for which there are now no treatments [11]. Wax moths are among these pests [11]. The term “wax moth” refers to a variety of moth species that attack, invade, damage, and destroy bee colonies and hive products [14]. Additionally, they are known as the wax (or web) worm, the bee (or wax) miller, or the bee moth [14]. The wax moth consists of two closely related species: the lesser wax moth (LWM), *Achrola grisella*, and the greater wax moth (GWM), *Galleria mellonella* (Lepidoptera: Pyralidae) [15]. Both are ubiquitous in honeybee colonies [16]. Globally the GWM is one of the most damaging and commercially important wax pests. To date, the economic losses ascribed to it have not been evaluated on a global scale [17]. However, the estimated loss due to GWM infestation is evaluated in the millions of dollars in many countries throughout the world such as the U.S., China, and European countries [13].

The GWM is one of a few insect pests that has adapted to living in honeybee hives. Although the adult moth is more visible to beekeepers, it is the larvae that are highly destructive to wax combs [18]. GWM larvae do not harm bees directly (i.e., they do not feed on any stage of the bee’s life stages), but they do attack beeswax combs, which are an important component of the honeybee nest [19]. In the comb midrib (the base of the cells), the larvae bore into the comb and develop a remarkable silken tunnel [20]. This damage is unique to the GWM and is known as galleriasis since they trap emergent bees, which therefore die of starvation [20]. Moreover, larvae of the GWM have been marked as potential vectors of many pathogens. For example, spores of *Paenibacillus* larvae were found in the larvae fecal pellets dropped in the silken tunnels, and black queen cell virus (BQCV) is linked with honeybee colony loss [17].

Little is known about the worldwide GWM distribution patterns [21], but various biotic and abiotic variables influence its occurrence and dispersion [21]. The GWM, like other honeybee pests, can thrive under certain bioclimatic conditions. Ecological niche modeling (ENM) tools can establish a statistical correlation between geographical changes in bioclimatic variables and the distribution of a certain species such as honeybee pests [22]. In the last two decades, a variety of modeling software based on various mathematical techniques has been developed to achieve this goal; however, the Maximum Entropy (Maxent) Model is the most successful and accurate [23,24,25]. Because of its outstanding predictive performance, Maxent modeling is very useful for predicting the impact of climate change on a variety of insect species, including honeybee pests [26,27].

Consequently, we aimed in this study to predict the global current and future distribution of the GWM using the species distribution modeling (SDM) approach. The outcomes of this study are extremely important and provide an urgent warning for beekeeping safety in various geographical areas.

## 2. Materials and Methods

### 2.1. Occurrence Data

**Figure 1:**
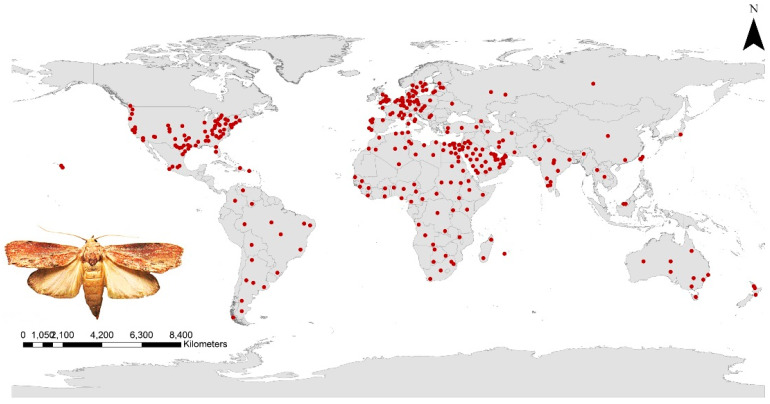
Distribution of the occurrence records used in studying the species distribution modeling of *Galleria mellonella*.

Approximations of greater wax moth occurrence have been reported on all continents except Antarctica, and almost all the available records were collected from the literature [28,29,30,31,32,33,34,35,36,37,38,39]. In addition, records from digital databases including the Global Biological Information Facility (GBIF.org (accessed on 3 January 2022): https://doi.org/10.15468/dl.hbhmdq), and the Centre for Agriculture and Bioscience International (CABI: www.CABI.org (accessed on 1 January 2022)) were taken into account. The occurrence data were subjected to three filtration steps to avoid any possible bias. First, duplicated records were removed [22]; second, records with high spatial uncertainty were eliminated; and third, to prevent redundant records, spatially rarefied occurrence data based on distance in ArcGIS (SDM Toolbox: SDM Tools; Universal Tools—Spatially rarefy occurrence data) were filtered. Finally, the remaining records with 313 points were transformed into comma-delimited (CSV) forms and used to predict the current and future worldwide distribution of the GWM (Figure 1).

### 2.2. Environmental Covariates

To carry out species distribution modeling (SDM), we obtained 19 bioclimatic variables (www.worldclim.org (accessed on 18 November 2021) having a spatial resolution of approximately 5 km^2^. Monthly temperature and rainfall readings collected from forecast stations between 1950 and 2000 were used to create these covariates.

For current bioclimatic data, 15 bioclimatic covariates were converted into ASCII format using ArcGIS v 10.7. Bioclimatic layers 8–9 and 18–19 were eliminated due to known spatial artifacts [22]. We applied the Pearson correlation coefficient to judge the correlation between each pair of covariates (r^2^ ≥ |0.8|) to reduce collinearity between variables [27,40]. This coefficient removed the correlation among the covariates through the function of SDM Tools in ArcGIS 10.7 (Universal tool; Explore climate data; Remove highly correlated variable) [40]. Based on this, only five bioclimatic covariates were selected to produce final models: annual mean temperature (Bio 1), mean diurnal range (Bio 2), annual temperature range (Bio 7), annual precipitation (Bio 12), and precipitation of the driest month (Bio 14).

For future bioclimatic data, we obtained parallel covariate datasets from (www.worldclim.org (accessed on 18 November 2021)), covering the two periods 2050 (average of estimates for 2041–2060) and 2070 (average of predictions for 2061–2080) [41]. These future data layers were also converted to ASCII format via ArcGIS v 10.7. Two representative concentration pathways (RCPs), 2.6 and 8.5 (https://www.worldclim.org/data/cmip6/cmip6climate.html (accessed on 18 November 2021)), were used to account for future GWM distribution. The RCPs were scenarios from the Coupled Model Intercomparing Project Phase 5 (CMIP5), which describes alternative dynamics for CO_2_ emissions and the resulting atmospheric concentration, the lowest anthropogenic radiative forcing level scenario (RCP 2.6), and high greenhouse gas emissions (RCP 8.5).

We used three general circulation models, or global climate models (GCMs), for each RCP in each time period for a total of 12 combinations (i.e., 3 GCMs × 2 RCPs × 2 time periods). The three GCMs are the Beijing Climate Center (BCC-CSM 1_1), the National Center for Atmospheric Research (CCSM4), and, the Meteorological Research Institute (MRI-CGCM3). These GCMs are part of the current GCM climate estimates in the IPCC’s Fifth Assessment Report. Finally, we obtained the mean predicted distribution of each RCP (2.6 and 8.5) for all three GCMs at each of the two periods, 2050 and 2070, to be easily compared with the current distribution of the GWM.

### 2.3. Modeling Approach

Among several software packages such as BIOCLIM, CLIMEX and GARP [42,43], Maxent has been used to predict the current and future global distribution of the GWM. Meanwhile, Maxent’s artificial intelligence of maximum entropy is often regarded as the most widely used software for simulating species distributions using presence-only data [44,45]. Maxent has outperformed other methods for estimating possible species distributions, regardless of the quantity or geographical range of species records, when compared to other software [45]. Thus, Maxent v 3.4.1 was used to model the current and future global distribution of the GWM. In our models, 75% of the occurrence records were used to train the model, whereas 25% of the records were used to test it [40]. The number of background points and iterations were set at 10,000 and 1000, respectively [46]. Furthermore, 10-fold cross-validation was used to repeat the process, which increased the model’s performance [46]. The habitat suitability regions of all the resultant models were classified into five classes––rare, low, medium, high, and very high––in Arc GIS through layer properties (Classified—Symbology—Natural Breaks (Jenks)) [27]. 

### 2.4. Model Evaluation

Model performance was estimated using the area under curve (AUC) of the receiver operating characteristics (ROCs), which ranged from 0 (random discrimination) to 1 (perfect discrimination) [27,47]. Models with AUC values less than 0.5 were considered poor-fitting, while those with values greater than 0.75 were considered well-fitting [47]. The accuracy of the projected models was further assessed using True Skill Statistics (TSS) [27,48], in which values ranged from 0 to 1. Positive values close to 1 indicated a good relationship between the predictive model and the distribution, whereas negative values close to 0 indicated a weak relationship [48].

## 3. Results

### 3.1. Modeling Performance

The AUC is one of the effective evaluation parameters of maximum entropy modeling and tends to be high with good modeling outputs. Here, the AUC equaled 0.8, indicated that the model had a considerable impact on the GWM environment (Figure S1). For a functional assessment of the model, TSS was used, and a value of 0.7 illustrated good map-producing quality. As a general rule, TSS values ≥0.5 are acceptable.

### 3.2. Contribution of Bioclimatic Variables

**Figure 2:**
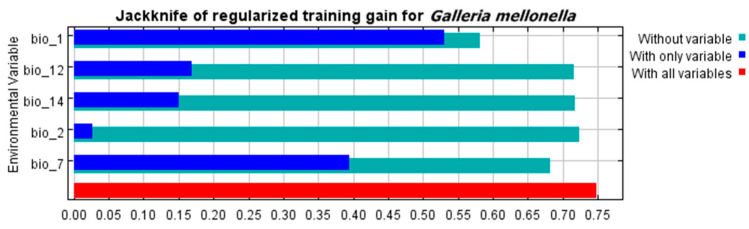
The jackknife test of the most important variables.

The contribution percentage of each bioclimatic variable of the predictive distribution model is illustrated using the jackknife test (Figure 2, Table 1). The results indicated the importance of temperature-related variables to GWM modeling. Annual mean temperature (bio_1) was the most effective climatological parameter that affected GWM distribution. In addition, the annual temperature range (bio_7) made a large contribution to the distribution of this pest. Annual precipitation (bio_12) came third among effective factors for pest allocation (Figure 2, Table 1). Meanwhile, according to the response curves of the most important environmental factors, the favorable annual mean temperature for the GWM ranged from 5 to 28 °C (Figures S2).

### 3.3. Predicted Current Potential Distribution of GWM

**Figure 3:**
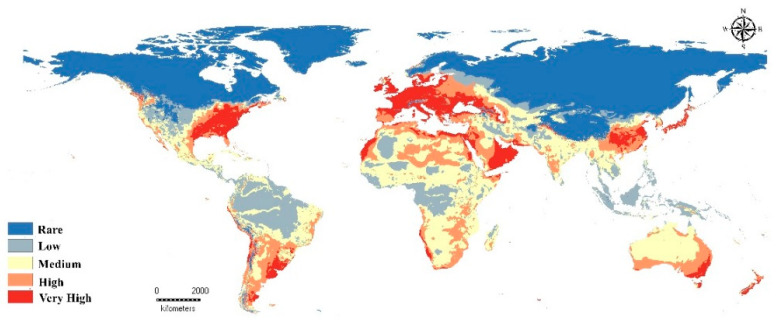
Current potential distribution of *Galleria mellonella*.

Based on distribution records and environmental covariates, the current model produced by Maxent agrees with the natural distribution range of the GWM (Figure 3). In Europe, great habitat suitability for the GWM puts Slovenia, Slovakia, France, Italy, Belgium, and Great Britain at high or very high risk. Africa, on the other hand, shows medium suitability throughout most of the continent with high and very high risks on the Mediterranean coast as well as the coasts of Morocco, Namibia, Western Sahara and parts of South Africa and the African horn. Further, in Asia, the risk is high through the southern part of the continent with very high risk in some parts of the Persian Gulf, China, India, Vietnam, Thailand and Japan (Figure 3); otherwise, the New World did not differ from the Old World in suitability for the GWM. Eastern North America, especially the U.S., appears to be at very high risk with some pockets of very good suitability through the western parts. The southwest coastal areas of South America, including Chile and the middle line of Latin America, as well as the eastern Atlantic coasts of Argentina and Brazil also have high-risk areas (Figure 3). The Australian eastern coast is also at very high risk while its mainland shows medium-to-high suitability. New Zealand appears to have high and very high suitability for such pests. The produced model clearly indicated the international distribution of the GWM (Figure 3).

### 3.4. The Predicted Future Potential Distribution of GWM in 2050 and 2070

**Figure 4:**
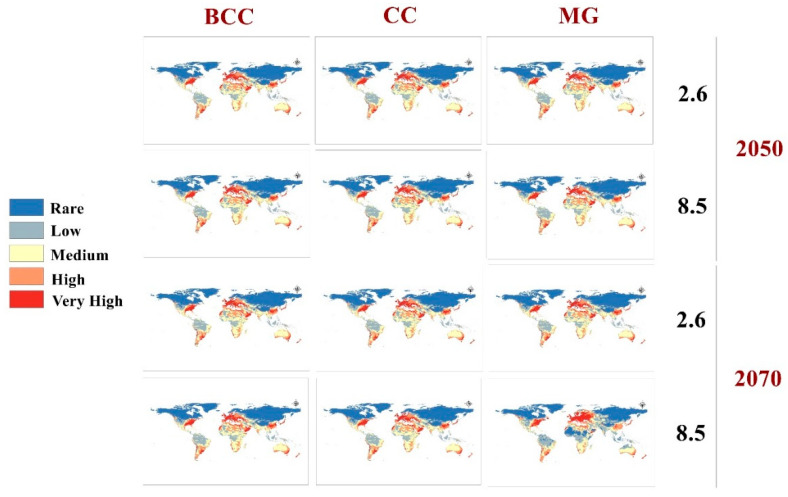
Predicted future distribution of *Galleria mellonella* under two RCPs (2.6 and 8.5) for three future scenarios (GCMs).

Three GCMs were used to evaluate the future status of the GWM during 2050 and 2070 using RCPs 2.6 and 8.5 (Figure 4). Future changes ranged from nonsignificant changes in the BCC 2.6 scenario to very clear changes in MG 8.5: a clearly visible loss in habitat suitability in tropical Africa and a gain in habitat suitability in northern Europe and North America (Figure 4).

**Figure 5:**
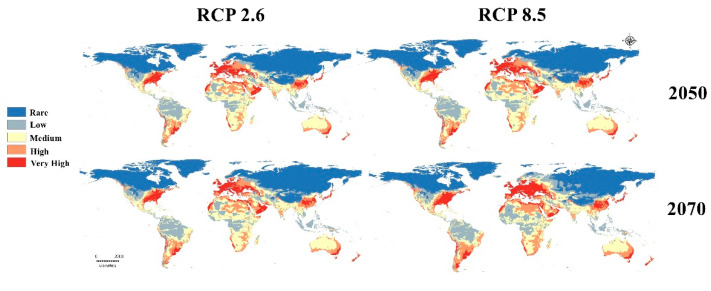
Predicted maps for the mean of three future GCMs using four RCPs scenarios: RCP 2.6 for 2050; RCP 8.5 for 2050; RCP 2.6 for 2070, and RCP 8.5 for 2070.

**Figure 6:**
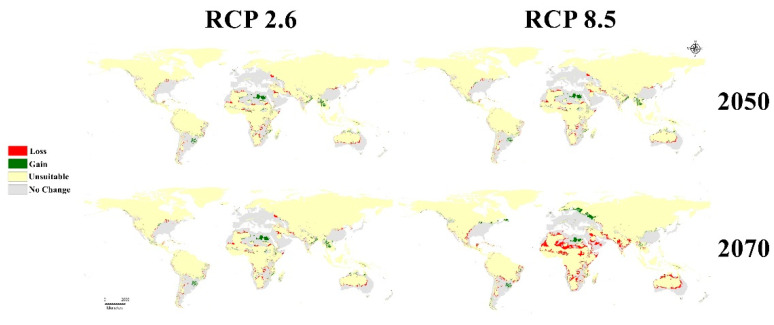
Calibration maps showing gain and loss in habitat suitability of *Galleria mellonella* through the four mean future scenarios against current status: RCP 2.6 for 2050; RCP 8.5 for 2050; RCP 2.6 for 2070, and RCP 8.5 for 2070.

The mean risk maps of the three GCMs for two different RCPs in 2050 and 2070 appear to summarize the level of changes to GWM risk due to global warming (Figure 5). For the optimistic climatic change prediction (2.6, 2050 RCP), the changes are very quiet and usually not significant on all continents (Figure 5 and Figure 6). Furthermore, for the worst climate change scenario (8.5, 2070 RCP) the insect loss has a very large range, especially in the subtropical region of Africa or Asia, and a clear range shift appears in northern Europe (Figure 6).

### 3.5. Two-Dimension Niche Analysis

**Figure 7:**
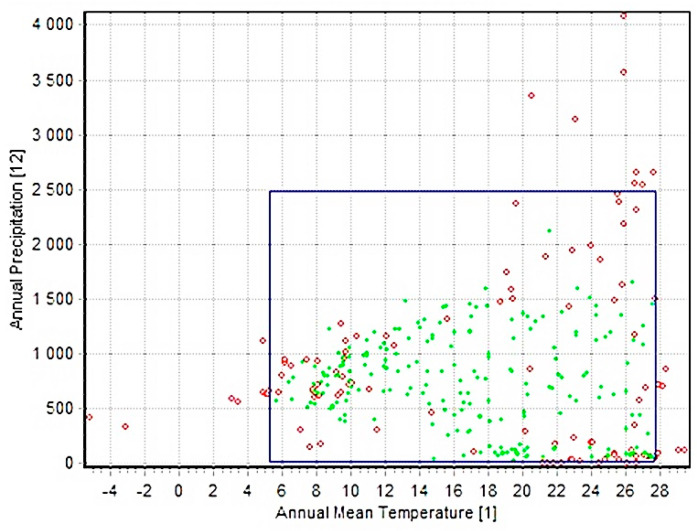
Two dimensional niche of *Galleria mellonella* between annual temperature (bio 1) and annual precipitation (bio 12).

The enveloped test was used to generate the two-dimension niche of the GWM for the most effective bioclimatic variables used in studying this pest: the annual mean temperature (bio_1) and the annual precipitation (bio_12) (Figure 7). The results indicated the impressive adaptability of this pest to different environmental conditions. Its annual mean temperature ranges from 5 to 28 °C, and the annual amount of rainfall ranges from 0 to 2500 mm. Such results give a great idea about the broad distribution of this notorious pest as it can live in very dry hot deserts and very cold rainy areas.

## 4. Discussion

Beekeeping is critical to the world’s food supply [21,22] as pollinators are very important for global food production and nutrition security [49,50,51]. Beekeeping also helps conserve natural resources, particularly in populations living near forests [52], and it provides a major source of national revenue through the sale of honey, wax, propolis, royal jelly, and bee venom [21,49,53,54]. 

Honey consumption has consistently increased over the past decades for two main reasons: an expanding global population, and a growing number of customers, notably from young people, who prefer natural foods [13]. Because of these factors, many countries cannot meet their honey need from domestic production alone and must import increasing amounts [13,21]. Furthermore, in some cases certain honey-importing countries require significant quantities of cheap honey from the international market so that it can be re-exported as locally produced honey [13,55]. The United States, Germany, Japan, the United Kingdom, and other European countries currently rank the highest in honey imports [13].

Factors like climate change, habitat fragmentation and loss, agriculture intensification, overdependence on agrochemicals, and, increasingly, viruses, pests, and diseases all pose threats to honeybee health, pollination, and associated livelihoods [20]. Pests are the most economically important because of their wide geographic distribution and ability to cause both direct (physical injury) and indirect (pathogen and disease vector) damage [21]. In bee health, it is critical to understand the elements that determine the multiplication and dissemination of honeybee pests on various spatial scales, as well as the level of risk [49]. As a result, enhanced pest diversity forecasting and accurate honeybee pest dispersion maps will provide the tools and information needed to combat honeybee pests, one of which is the GWM, considered to be one of the most distributed and destructive [13,20,21].

Our present work is the first study to assess the global warming and climate change effects on the global distribution of the GWM using the robust predictive power of Maxent. The projected habitat suitability of our Maxent model coincided closely with the actual occurrence of GWM records with a high AUC value, implying a close association between the model and the species’ ecology. Furthermore, the TSS value of 0.7 indicated that the model predictions and the dispersion of the pest were in perfect accord.

The most important characteristic influencing GWM distribution, according to previous studies, is temperature [20,21]. According to our model, ambient temperature was the most effective bioclimatic variable affecting GWM distribution in the jackknife test. The annual mean temperature (Bio 1) and temperature annual range (Bio 7) contributed 64.2 and 19%, respectively, in GWM distribution. The other bioclimatic factors, according to the jackknife test, were annual precipitation (Bio 12), precipitation of driest month (Bio 14), and mean diurnal range (Bio 2) (Figure 2). Moreover, the two-dimensional niche analysis confirmed temperature as a key covariate affecting GWM distribution (Figure 7).

The current model for the potential distribution of the GWM is closely associated with present-day distributions (Figure 3). Almost all of mainland Europe––north to south and east to west––showed very high suitability for the GWM. Hot-spot lands of the honeybee industry, e.g., Slovenia and Slovakia, showed very high suitability. Only Belarus, Ukraine, and Portugal showed high suitability. European markets are characterized by the high quality of honey and its products [13,56], so the very high suitability for the GWM has a negative impact on honeybee markets. Honey imports into the EU have increased at an average rate of 10,284 tons per year over the last 15 years, with China being the primary source of that growth [13]. The prices EU countries pay for Chinese honey are highly disparate, which could be due to the differing importer quality standards [57]. Although a high price does not guarantee honey purity, cheap honey does have a larger chance of being tampered with [58]. As a result, the import price can serve as the first indicator of poor honey quality, prompting the need for additional research into the product’s purity [57,58].

In Asia, our current predictive model showed high and very high suitability in the Persian Gulf region, India, China, and Japan, which is one of the highest importing countries for honeybee products [13]. The effect of very high GWM suitability is especially maximized in China, one of the most important honeybee product exporting countries [13]. Annual Chinese exports equal approximately 128,330 tons followed by India at 35,793 tons, which also showed high suitability [59].

In the New World, our current model showed areas with high and very high suitability. The U.S., Mexico, Argentina, Brazil, and Chile showed high suitability for the GWM. The market for honey imports in the United States has been steadily increasing [60], and one reason is because of destructive pests like the GWM and *Varroa* mite [61]. Domestic U.S. honey production fell by 705 tons per year in recent years, while honey imports climbed by 6,956 tons per year [60]. In 2017, domestic honey production only met 25% of U.S. demand. Following the “honeygate” investigation into the illegal importation of honey from China, the pattern of honey imports changed [13]. Meanwhile, Argentina, Brazil, and Chile are the main exporters of honeybee products with 81,183; 24,203; and 7,137 tons, respectively [13,60]. Both Australia and New Zealand were included in the list of the 25 honey-exporting countries in 2016 [13]. Furthermore, the eastern coast of Australia and the mainland of New Zealand showed very high suitability for the GWM, which threatens their honeybee products industry.

In Africa, generally, the suitability of the GWM is considered to be medium risk due to high temperature [21], but tropical countries, the Horn of African and South Africa showed very high suitability. The economy of the honeybee market and its products in Africa are not clear [52], which may be due to poor vegetation and the low value of African honey [21,52]. Meanwhile, our predictive models suggest that the GWM has a destructive effect in countries like Egypt, Kenya, Morocco, and South Africa, which have very high suitability.

Future predictive models illustrate that the pest will have a wider distribution range. Three GCMs under two RCPs (2.6 and 8.5) for 2050 and 2070 were used to evaluate the global future status of the GWM (Figure 4, Figure 5 and Figure 6). From the trusted maximum entropy implemented in Maxent, the 12 future scenarios confirmed the dangerous status of the GWM throughout the world (Figure 4 and Figure 5), and the near and distant future threats to honeybee products industries (Figure 4). Our predictive future models in the three GCMs in 2050 and 2070 clarified that hot-spot European countries for honeybee products (e.g., Ukraine, Germany, France, Italy). Belgium, Slovenia, and Slovakia will suffer from very high GWM suitability [62] (Figure 5). Moreover, the GWM showed high and very high suitability in future models for China, India, Vietnam, and Thailand, which are considered to be the highest Asian exporters of honeybee products [13,59,62]. Similarly, in the Americas, the U.S., Mexico, Argentina, Brazil, Chile, and Cuba will have a very high suitability (Figure 4 and Figure 5). Likewise, the future predictive models accounted for the invasion of the GWM to the mainland of Australia and New Zealand. Finally, in Africa, our future models predict habitat loss for the GWM in tropical and subtropical Africa due to temperature increases.

Our research contributes to a better understanding of the current and future status of the GWM around the world. The models developed in this study analyzed the effect of climate change on the existing and future distribution of the GWM using just climatological factors. For this objective, several papers only used climate factors [22,27,40]. Considering other environmental variables, such as human population, land cover, vegetation index, and host animal distribution, could help to improve them. However, the lack of future data on these variables may restrict their utility in researching the impact of climate change on present distribution models.

## 5. Conclusions

The greater wax moth (GWM) targets wax combs either inside or outside beehives and is considered among the most common apiculture pests. The economic damage from the GWM to the beekeeping industry has not been widely elevated, but it is expected to be high. Herein, ecological niche modeling was used to evaluate habitat suitability for the GWM at two different times. The models showed high performance with highly accurate outputs. The ambient temperature factor was the most effective bioclimatic variable that affected GWM distribution. In addition, the annual mean temperature and annual temperature annual range contributed 64.2 and 19% respectively to the GWM distribution. The resultant models highlighted a very high habitat suitability for the GWM in countries with high beekeeping activity. Developing prompt monitoring and control strategies is essential because this pest adapts to a wide range of environmental conditions.

## Figures and Tables

**Table 1:** Relative percentages of bioclimatic variables used in Maxent to model the current and future habitat suitability of the Greater Wax Moth (GWM), *Galleria mellonella*.

Bioclimatic Variables	Description	Contribution Percentages
Bio 1	Annual Mean Temperature	64.2%
Bio 7	Temperature Annual Range	19%
Bio 12	Annual Precipitation	7.2%
Bio 14	Precipitation of Driest Month	5.6%
Bio 2	Mean Diurnal Range (Mean of monthly max temp—min temp)	4.1%

## Data Availability

The data presented in this study are available in the article.

## Supplementary Materials

The following supporting information can be downloaded at: https://www.mdpi.com/article/10.3390/insects13050484/s1, Figure S1: The receiver operating characteristic (ROC) curve for Greater wax moth (GWM), *Galleria mellonella*; Figure S2: Response curves of the most relevant environmental factors affecting the distribution of the greater wax moth (GWM), *Galleria mellonella*; the shown values are average of ten replicate runs.
