# Supplementary material for: Modeling the Potential Global Distribution of Honeybee Pest, Galleria mellonella under Changing Climate
Source: Insects. 2022 May 22;13(5):484. doi: 10.3390/insects13050484 (PMC9143048; doi:10.3390/insects13050484)

Figure S1: The receiver operating characteristic (ROC) curve for Greater wax moth (GWM), *Galleria mellonella*

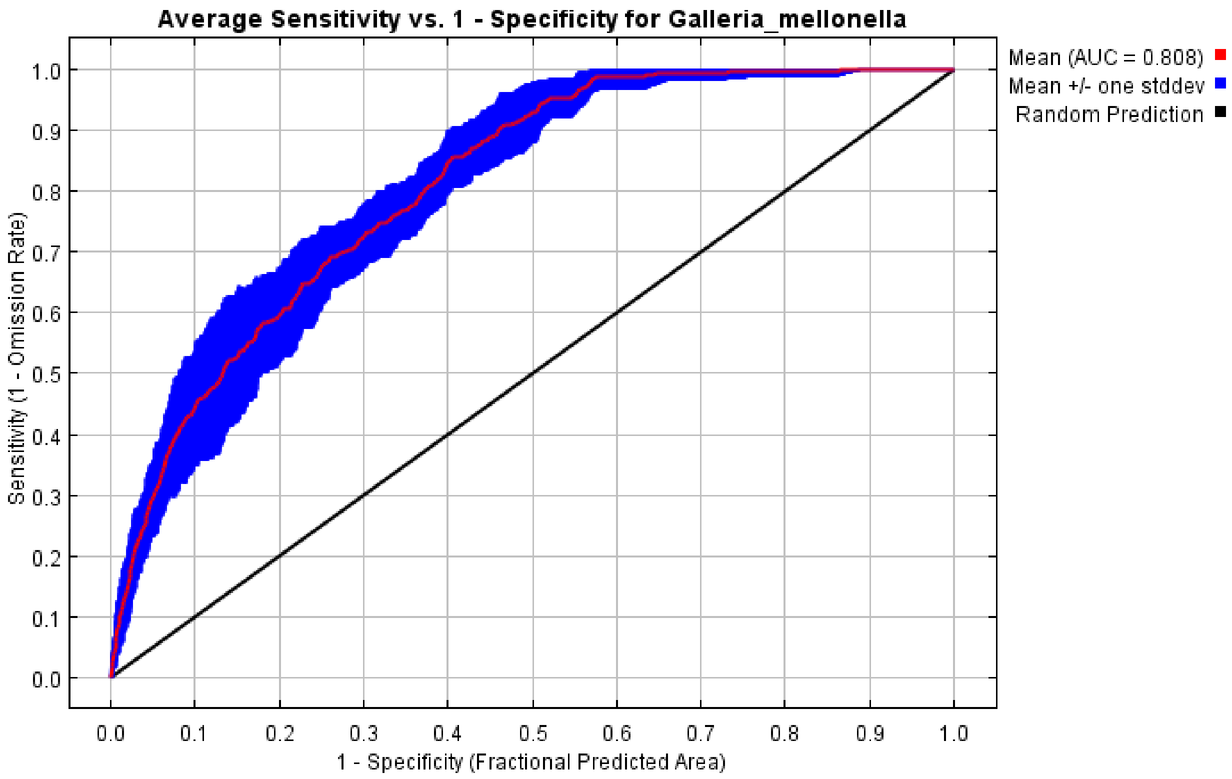

Supplement: Supplementary file 1 [file insects-13-00484-s001.zip › Figure S1.pdf]
