# Supplementary material for: Modeling the Potential Global Distribution of Honeybee Pest, Galleria mellonella under Changing Climate
Source: Insects. 2022 May 22;13(5):484. doi: 10.3390/insects13050484 (PMC9143048; doi:10.3390/insects13050484)
Supplement: Supplementary file 1 [file insects-13-00484-s001.zip › Figures S2.pdf]

Figures S2: Response curves of the most relevant environmental factors affecting the distribution of the greater wax moth (GWM), *Galleria mellonella*; the shown values are average of ten replicate runs.

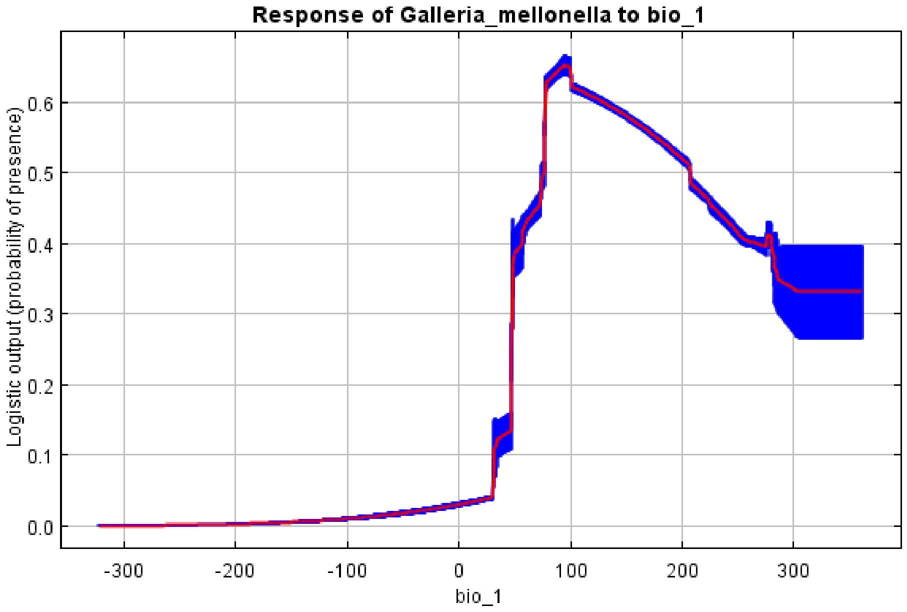

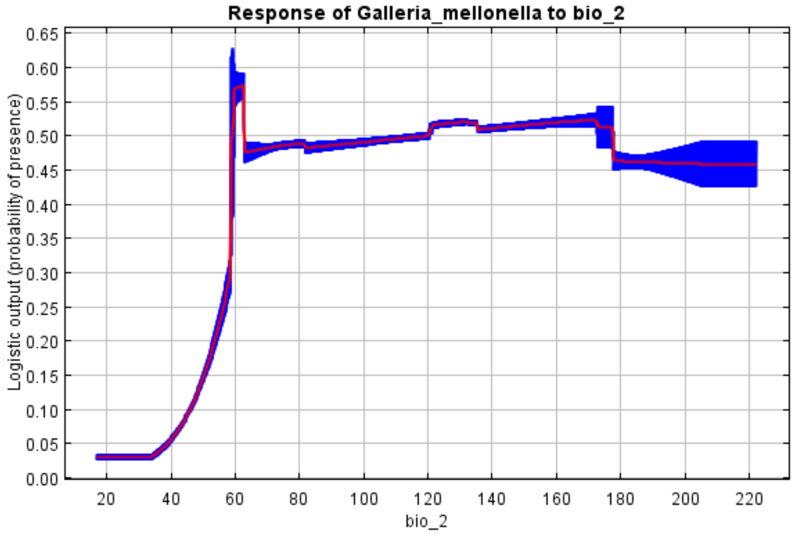

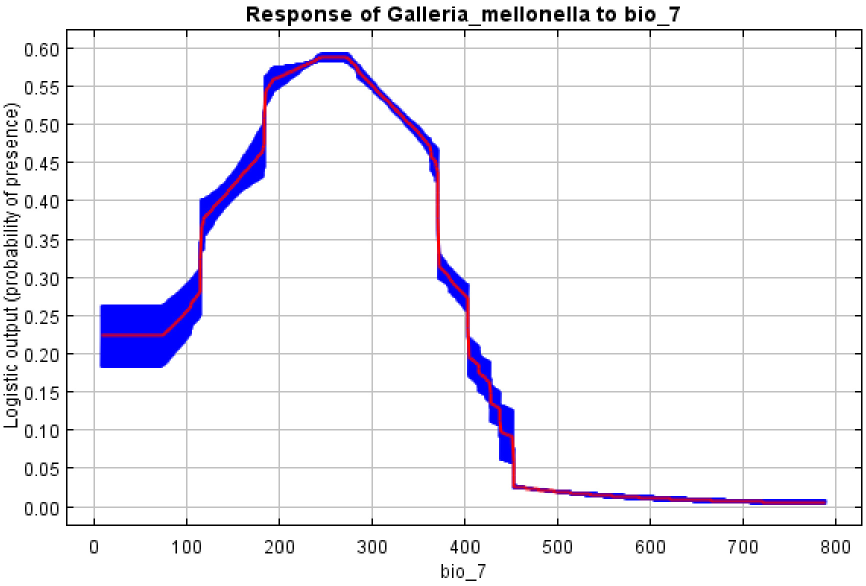

Response of Galleria_mellonella to bio_7
Logistic output (probability of presence)
0.60
0.55
0.50
0.45
0.40
0.35
0.30
0.25
0.20
0.15
0.10
0.05
0.00
0
100
200
300
400
500
600
700
800
bio_7

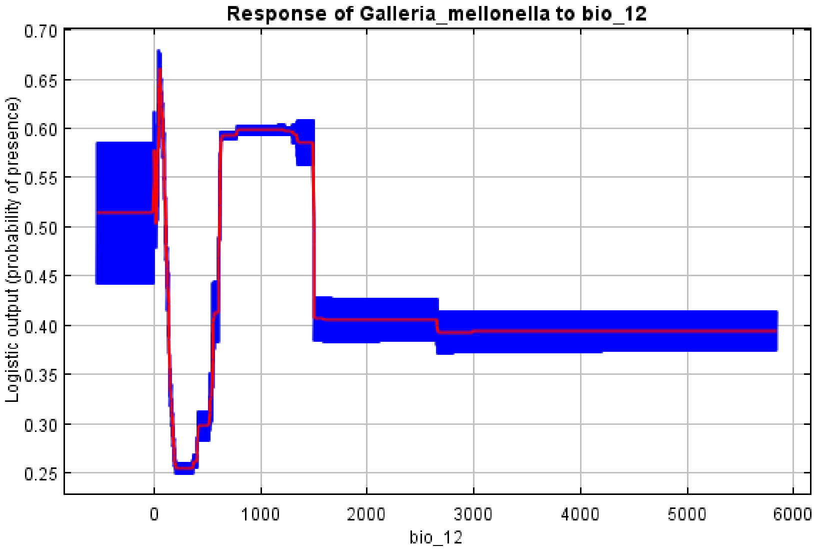

Response of Galleria_mellonella to bio_12
Logistic output (probability of presence)
0.70
0.65
0.60
0.55
0.50
0.45
0.40
0.35
0.30
0.25
0
1000
2000
3000
4000
5000
6000
bio_12

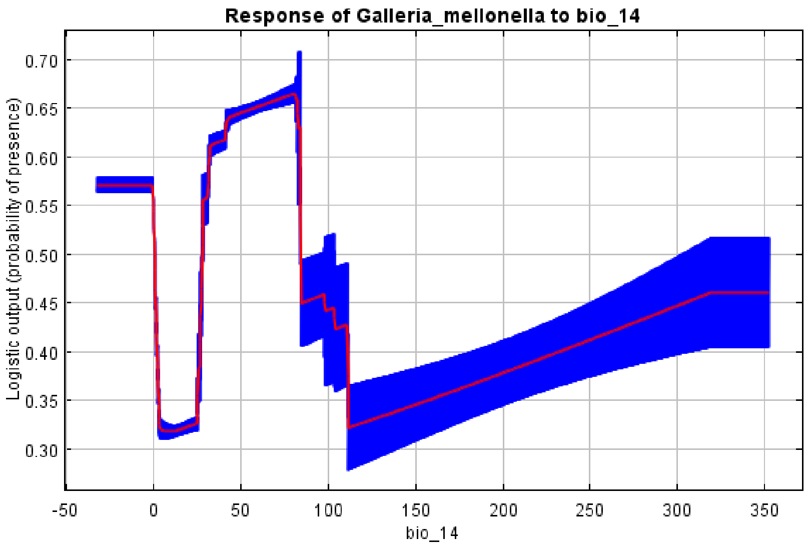
Response of Galleria_mellonella to bio_14
Logistic output (probability of presence)
0.70
0.65
0.60
0.55
0.50
0.45
0.40
0.35
0.30
-50
0
50
100
150
200
250
300
350
bio_14
